# Suvorexant Poisoning in a Patient With Cirrhosis and Renal Failure

**DOI:** 10.7759/cureus.14329

**Published:** 2021-04-06

**Authors:** Hiroshi Ito, Yasuhiro Ogawa, Nobutake Shimojo, Satoru Kawano

**Affiliations:** 1 Division of Hospital Medicine, University of Tsukuba Hospital, Tsukuba, JPN

**Keywords:** suvorexant poisoning, hypnotic

## Abstract

Suvorexant is a novel hypnotic that acts as an orexin-1 receptor and orexin-2 receptor antagonist. Owing to its safety and tolerability, suvorexant has recently become widely used. However, little is known about the presentation of suvorexant poisoning. Here, we describe an 83-year-old man with cirrhosis and renal failure, who had taken 270 mg of suvorexant at the same time. After the overdose, he did not develop any symptoms other than prolonged drowsiness. He was successfully treated with supportive therapy alone. This is the first report describing suvorexant poisoning. Further reports should be accumulated to determine whether patients with suvorexant poisoning present with mild symptoms without intensive treatment.

## Introduction

Suvorexant is a novel hypnotic that was first approved in the United States in 2014 [1]. Unlike other hypnotics, such as benzodiazepines, suvorexant acts as an orexin-1 receptor and orexin-2 receptor antagonist. Suvorexant has been known to be generally safe and well-tolerated when used in a standard dose [2]. Besides, its preventive effect on delirium has recently been reported, which seems to have accelerated its use [3]. However, little is known about the presentation of suvorexant poisoning. Here, we report a case of suvorexant poisoning in a patient with cirrhosis and renal failure. The patient did not present with any symptoms other than prolonged drowsiness, and supportive therapy was sufficient.

## Case presentation

An 83-year-old Japanese man was admitted to the emergency department because of suvorexant poisoning. He had taken 270 mg of suvorexant 12 hours before admission at the same time because he could not endure pruritus resulting from cirrhosis and renal failure. After intoxication, he did not wake up until two hours before admission, which means he slept for about 10 hours. His medical history included hypertension, type 2 diabetes, hepatic cell carcinoma, nonalcoholic steatohepatitis, and insomnia. He also had chronic renal failure secondary to renal sclerosis, but he had not received renal replacement therapy. He had undergone bilateral cataract surgery. Owing to these comorbidities, he was taking multiple medications (Table 1).

**Table 1:** The patient’s comorbidities and medications on admission.

Comorbidity	Medication
Hypertension	Amlodipine (10 mg/day)
Type 2 diabetes	Atorvastatin (10 mg/day)
Dyslipidemia	Febuxostat (20 mg/day)
Hyperuricemia	Furosemide (40 mg/day)
Renal sclerosis, renal failure	Calcium Polystyrene Sulfonate (25 g/day)
Hepatic cell carcinoma	Sodium bicarbonate (2,000 mg/day)
Cataract	Sodium ferrous citrate (100 mg/day)
Gastroesophageal reflux disease	Roxadustat (150 mg/week)
Pruritus	Lactulose (18 g/day)
Insomnia	Ursodeoxycholic acid (300 mg/day)
	Lansoprazole (15 mg/day)
	Rebamipide (300 mg/day)
	Epinastine hydrochloride (20 mg/day)
	Suvorexant (15 mg/day)

**Figure 1:**
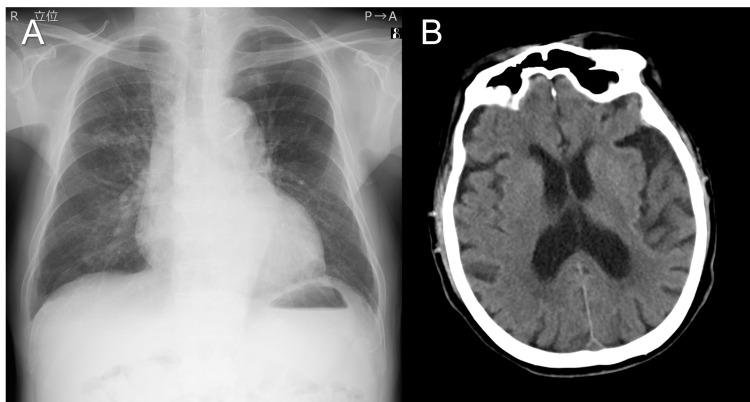
Imaging studies on admission. (A) The chest radiograph revealed no abnormalities. (B) The computed tomographic images of the brain showed no abnormalities.

**Figure 2:**
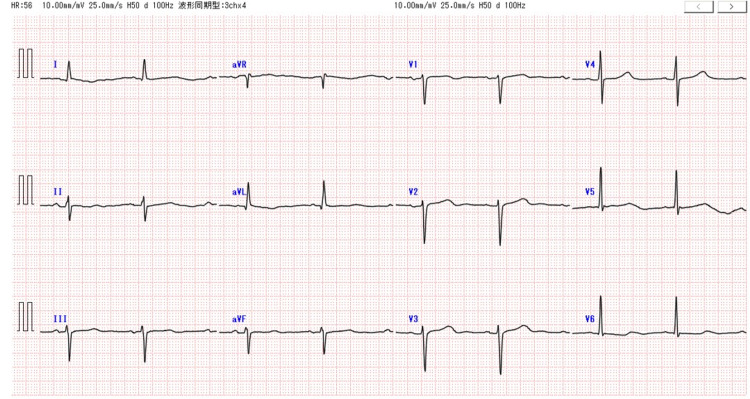
The electrocardiogram before and on admission. The electrocardiogram on admission was notable for QT prolongation and bradycardia but did not present any other remarkable changes.

He was alert on admission, and his vital signs showed an axillary body temperature of 36.7°C, a heart rate of 60 beats per minute, a blood pressure of 161/58 mmHg, oxygen saturation of 96% on room air, and a respiratory rate of 12 breaths per minute. He was alert and not agitated or sedated. He did not present flapping tremors. Physical examination revealed no remarkable neurologic deficits other than miosis and loss of the pupillary light reflex; however, these symptoms were considered to be due to his previous cataract surgery. He developed neither hyperhidrosis nor anhidrosis. Both laboratory and radiographic evaluations were unremarkable (Tables 2, 3 and Figures 1A, 1B). The urine drug test findings were negative. The electrocardiogram was notable only for QT prolongation and bradycardia (Figure 2).

**Table 2:** The arterial blood gas test on admission. The results of the blood gas test were unremarkable. PCO_2_, partial pressure of carbon dioxide; PO_2_, partial pressure of oxygen

	Reference value	Day 1
pH	7.35–7.45	7.414
PCO_2_ (mmHg)	35–48	39.6
PO_2_ (mmHg)	80–100	78.9
Bicarbonate (mmol/L)	22.2–28.3	24.9
Sodium (mmol/L)	136–145	136
Chloride (mmol/L)	98–107	101
Potassium (mmol/L)	3.5–4.5	3.7
Calcium (mmol/L)	1.15–1.33	1.09
Glucose (mg/dL)	65–95	108
Lactate (mmol/L)	0.56–1.39	0.5
Plasma osmolality (mOsm/L)	275–290	279
Base excess (mmol/L)	-3.2 to 1.8	0.8

**Table 3:** The blood tests before and after admission. Lactate dehydrogenase and C-reactive protein were mildly elevated.

	Reference value	Day -3	Day 1	Day 2	Day 4
White blood cell (/μL)	4,000–9,000	5,300	4,100	4,100	4,900
Hemoglobin (g/dL)	14.0–18.0	6.4	7.1	6.2	8.1
Mean corpuscular volume (fL)	80–100	86.5	87.8	87.7	84.7
Platelet (/μL)	150,000–350,000	181,000	208,000	187,000	183,000
Aspartate transaminase (U/L)	8–38	13	15	15	15
Alanine transaminase (U/L)	4–44	9	10	8	9
Lactate dehydrogenase (U/L)	124–222	209	225	189	210
Alkaline phosphatase (U/L)	38–113	121	119	102	111
γ-glutamyl transpeptidase (U/L)	12–63	37	37	30	30
Total bilirubin (mg/dL)	0.3–1.2	0.2	0.2	0.2	0.4
Sodium (mEq/L)	135–147	133	140	140	139
Chloride (mEq/L)	98–108	95	100	102	104
Potassium (mEq/L)	3.6–5.0	4.3	4	4.2	4.2
Urea nitrogen (mg/dL)	8.0–20.0	86	86	86.2	83.2
Creatinine (mg/dL)	0.61–1.04	8.36	8.35	8.35	7.67
Amylase (U/L)	40–126			79	89
Creatine kinase (U/L)	63–257		99	101	69
Calcium (mg/dL)	8.6–10.1		8.1	7.9	7.9
Inorganic phosphorus (mEq/L)	2.7–4.5		5.1	5.5	4.4
C-reactive protein (mg/dL)	0.0–0.2	0.96	1.68	1.91	1.77
Prothrombin time (%)	80–120		106	101	107

**Figure 3:**
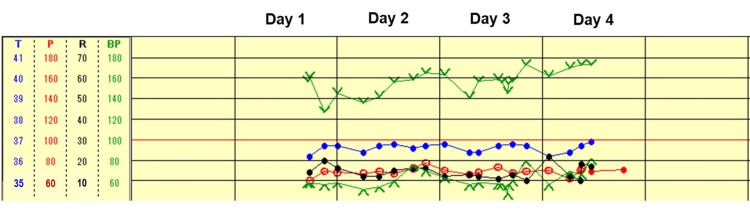
The patient’s vital signs during hospitalization. T, axillary body temperature; P, pulse rate; R, respiratory rate, BP, blood pressure

After admission, he received supportive therapy using half-normal saline in 5% dextrose. He received four units of red blood cell transfusion for anemia at two units per day on days 2 and 3. This anemia had been present before admission and was thought to result from renal failure, but his fecal occult blood test was positive. He then underwent upper gastrointestinal endoscopy, and no sign of active bleeding was found. He did not develop additional symptoms afterward and started oral intake. During his hospital stay, his vital signs were stable (Figure 3), and he was discharged home on day 5. At his most recent visit, 50 days after discharge, he was asymptomatic and doing well.

## Discussion

We reported a case of suvorexant poisoning. The patient presented no remarkable symptoms other than prolonged drowsiness, and he was treated successfully with supportive therapy alone.

Somnolence, fatigue, abnormal dreams, and dry mouth have been reported as adverse effects of suvorexant, but it has also been known to be safe and well-tolerated [2]. Owing to its safety, the expectations for suvorexant are in terms of its use as a benzodiazepine-sparing hypnotic agent. Overdoses in suicide attempts are unavoidable challenges associated with any hypnotic agent. For example, cases of ramelteon overdose, as well as benzodiazepine overdose, have recently been reported [4]. In this context, suvorexant would also be used for suicide attempts. However, to our knowledge, this is the first report describing suvorexant poisoning. Our patient did not present with specific symptoms other than prolonged drowsiness, and there was no toxidrome [5]. Our patient presented with miosis and no pupillary light reflex, but this was because he had undergone cataract surgery and it seemed irrelevant to the suvorexant poisoning. There was a possibility that his prolonged drowsiness had resulted from his multiple comorbidities. However, his clinical symptoms occurred acutely, and it seemed unlikely that these symptoms were due to his comorbidities, including cirrhosis, renal failure, and baseline anemia. Besides, laboratory data did not show evidence of the worsening of these comorbidities.

He recovered consciousness no more than 10 hours after intoxication despite having cirrhosis and renal failure. Serum suvorexant concentration has been known to increase dose-dependently, and it has a half-life period of approximately 12 hours [6]. Although this half-life period may seem relatively long for a hypnotic agent, this agent does not appear to have significant next-day residual effects. One hypothesis is that circadian variation of orexin levels may play a role in determining the residual effects. Our patient awoke around noon, and naturally increasing orexin levels may have counteracted the suvorexant residual effects [7]. Unfortunately, suvorexant measurement has not been established in Japan, and we could not measure the serum suvorexant levels in our case.

Suvorexant poisoning was successfully managed with supportive therapy alone in our case. Due to the mildness of his symptoms, he did not receive mechanical ventilation and renal replacement therapy during his hospital stay. Hypnotic poisoning, such as benzodiazepine poisoning, rarely causes significant toxicity [8]. However, patients with hypnotic poisoning sometimes suffer severe toxicity presenting as a stuporous or comatose state, requiring intensive treatment. This risk for severe toxicity often perplexes physicians when prescribing hypnotics for patients with high suicide risk. In such situations, suvorexant may be used as a relatively safe alternative for conventional hypnotics.

## Conclusions

Our patient with suvorexant poisoning did not present with specific symptoms other than prolonged drowsiness, and he was successfully treated with supportive therapy alone. Considering these points, suvorexant seems relatively safe in insomniac patients with a high risk of intoxication and suicide. However, this is the only published report on suvorexant poisoning, and our report has a limitation of potentially confounding comorbidities of the case. Thus, little is still known about this situation. Further reports should be accumulated to determine whether patients with suvorexant poisoning present with mild symptoms without intensive treatment.
